# Monensin Suppresses Multiple Features of House Dust Mite-Induced Experimental Asthma in Mice

**DOI:** 10.1007/s10753-024-02090-7

**Published:** 2024-07-03

**Authors:** Venkata Sita Rama Raju Allam, Srinivas Akula, Ida Waern, Sowsan Taha, Sara Wernersson, Gunnar Pejler

**Affiliations:** 1 Department of Medical Biochemistry and Microbiology, Uppsala University, Uppsala, Sweden; 2 Department of Anatomy, Physiology and Biochemistry, Swedish University of Agricultural Sciences, Uppsala, Sweden

**Keywords:** Asthma, Inflammation, House dust mite, Monensin, Mast cells

## Abstract

Despite intense efforts to develop efficient therapeutic regimes for asthma, there is a large demand for novel treatment strategies in this disease. Here we evaluated the impact of monensin, a drug with potent anti-mast cell effects, in a mouse model of asthma. Allergic airway inflammation was induced by sensitization of mice with house dust mite (HDM) antigen, and effects of monensin on airway hyperreactivity and inflammatory parameters were studied. Following intraperitoneal administration, monensin did not suppress airway hyperreactivity but was shown to have anti-inflammatory properties, as manifested by reduced eosinophil- and lymphocyte infiltration into the airway lumen, and by suppressed inflammation of the lung tissue. After intranasal instillation, monensin exhibited similar anti-inflammatory effects as seen after intraperitoneal administration. Moreover, intranasally administered monensin was demonstrated to suppress goblet cell hyperplasia, and to cause a reduction in the expression of genes coding for key inflammatory markers. Further, monensin blocked mast cell degranulation in the airways of allergen-sensitized mice. Together, this study reveals that monensin has the capacity to suppress key pathological events associated with allergic airway inflammation.

## Introduction

Asthma is a multifactorial disease of the airways, afflicting around 300 million people world-wide, and is characterized by airway narrowing leading to breathing difficulties, airway inflammation and extensive airway remodeling [1–7]. Altogether, these features have a far-reaching impact on afflicted people’s quality of life, and asthma exacerbations can even have fatal consequences. Based on this, there is a large need for effective regimes for the therapeutic intervention of asthma. Current treatment of asthma includes the usage of corticosteroids, bronchodilators, leukotriene receptor antagonists, as well as various biologicals such as anti-IL-5, anti-IL-4/13 and anti-IgE monoclonal antibodies [7, 8]. However, despite this broad panel of available treatment options, asthma control, in particular for severe asthma, is still not satisfactory, with many patients responding poorly to currently available medications. Moreover, treatment with biologicals is associated with high costs for the health care systems. Hence, there is a continued demand for improved anti-asthma therapy.

Previous research has established a key role for mast cells (MCs) as effector immune cells in asthma [9–13]. MCs are characterized by a high content of acidic secretory granules, and these are filled with large quantities of pro-inflammatory mediators such as histamine, cytokines and various proteases [14, 15]. When MCs are activated, e.g., by IgE receptor crosslinking, these mediators are released to the environment. Moreover, MC activation will typically result in *de novo* synthesis of additional pro-inflammatory compounds, the latter including prostaglandins, leukotrienes and a range of cytokines and chemokines [16]. Altogether, MC activation can thus lead to a massive release of numerous pro-inflammatory compounds, potentially leading to a powerful inflammatory reaction.

Based on the known contribution of MCs to asthma, an emerging concept for the treatment of this disease is to target MCs [17–19]. To approach this, we previously screened the Prestwick compound library, a drug library encompassing ~ 1200 approved drugs [20], for compounds having anti-MC properties. This led to the discovery that monensin, an antibiotic which is used for the treatment of coccidiosis, had potent anti-MC activity. Mechanistically, we found that monensin targeted the MC secretory granules, thereby inducing granule permeabilization leading to the leakage of acidic granule content into the cytosol [21]. In a follow up study, we demonstrated that also eosinophils are sensitive to monensin [22].

Considering the established role of MCs and eosinophils in asthma, monensin could thus have potential anti-asthmatic properties. Indeed, it was shown recently that monensin ameliorated multiple features of allergic airway inflammation in a guinea pig model, and it was also shown that monensin had the capacity to block airway narrowing *ex vivo* in human lung tissue specimens subjected to IgE-mediated activation [23]. Here, we extended the concept of using monensin as an anti-asthma drug by evaluating the compound in an established and physiologically relevant mouse model for asthma, based on sensitization with house dust mite (HDM) antigen. We show that monensin has the capacity to suppress airway inflammation in response to HDM sensitization. Hence, these findings reinforce the potential usefulness of monensin as an anti-asthmatic drug.

## Materials and Methods

### Animals

Female BALB/c mice (8 – 9 weeks of age) were purchased from Taconic Biosciences (Lille Skensved, Denmark). All procedures were performed at the Swedish University of Agricultural Sciences animal facility under protocols compliant with the EU Directive 2010/63/EU for animal experiments and approved by the local ethical committee (Uppsala djurförsöksetiska nämnd; Dnr 5.8.18–12873/2019). Mice were acclimatized for one week prior to the experiment. Mice were lightly anaesthetized with isoflurane using a portable isoflurane vaporizer. Anaesthetized mice were instilled with 10 μg of house dust mite (HDM; *Dermatophagoides pteronyssinus*, CiteQ BV, Groningen, The Netherlands; endotoxin content: 4600 EU/mg extract) extract reconstituted in 30 μl PBS intranasally twice a week for three weeks. Control mice received 30 μl PBS via the intranasal route. Monensin (Sigma-Aldrich; St Louis, MO) was administered either intraperitoneally (i.p.; 1 mg/kg in 4% ethanol in PBS) or intranasally (0.25 mg/kg in 4% ethanol in PBS or 1 mg/kg in 16% ethanol in PBS) or vehicle (4% ethanol in PBS for the i.p. route; 4 or 16% in PBS for the intranasal route). Monensin was administered thirty minutes before each PBS and/or HDM instillation. Control experiments showed that single i.p. administration of monensin or its vehicle (100 µl of 4% ethanol in PBS) showed no overall toxicity to the animals (after 24 h), as judged by monitoring of physical activity and behavioral changes assessed according to guidelines at the animal facility. Likewise, intranasal administration of monensin or its respective vehicles (4% ethanol in PBS or 16% ethanol in PBS, respectively) did not produce any detectable physical or behavioral changes. Further, intranasal administration of monensin or its respective vehicles did not result infiltration of inflammatory cells into the airway lumen, as assessed by assessment of cells recovered in bronchoalveolar lavage (BAL) fluid at baseline conditions (24 h after administration in the mice).

### Measurement of Airway Hyperreactivity

Airway hyperreactivity (AHR) in response to 0 – 50 mg/mL methacholine was assessed by using a Buxco small ventilator (Buxco® FinePointe RC, Winchester, UK), as described [24, 25]. Airway hyperreactivity was assessed by measuring lung resistance in response to increasing doses of metacholine. Lung compliance, i.e., a measure of the lung tissue elasticity, was also assessed.

### Bronchoalveolar Lavage

For bronchoalveolar lavage (BAL), cannulated lungs were lavaged twice with 0.5 mL of sterile Hanks Balanced Salt Solution (HBSS). The collected BAL fluid was centrifuged at 600 × g for 10 min (4 °C). Cell pellets were resuspended in 1 mL sterile HBSS for enumeration of total and differential cell counts. For total cell counts, a hemocytometer was used. For differential leukocyte counts, cytospin slides were prepared by centrifugating 100 µl cell suspensions onto glass slides (26 × g; 5 min using a cytospin centrifuge). Cells were stained with May Grünwald/Giemsa; a minimum of 200 cells per slide were counted.

### Lung Histology

Lung histology was assessed by haematoxylin and eosin (H&E) staining; airway inflammation was monitored by a tissue scoring protocol [24, 26]. Goblet cell hyperplasia and mucus production, as well as smooth muscle cell layer thickness was visualized by periodic acid-Schiff (PAS) staining [24]. For assessment of smooth muscle layer thickness, similar-sized cross-sectioned bronchi with intact structure were analyzed in all mice. The analyses were conducted on larger bronchi, which represent the major site of mast cell location. For each mouse, we analyzed one such bronchus, by calculating the average thickness from a total of 20 measurements evenly spread around the airway smooth muscle layer. The degree of mast cell degranulation was given a score based on the number of degranulated mast cells per lung tissue slide as follows: no degranulated cell (score 0), 1 degranulated cell (score 1), 2–3 degranulated cells (score 2), and > 3 degranulated cells (score 3) [27]. All lung tissue sections were analyzed blindly.

### Quantitative RT-PCR Analysis

Quantitative RT-PCR (qPCR) analysis was performed as described [24]. The primers for CCL11, CCL9 and Clec7a/Dectin1 were validated primers purchased from BioRad (Hercules, CA). The other primers were validated in house, with the following sequences:

IL-13, forward: 5′-AGGAGCTTATTGAGGAGCTGA-3′; IL-13, reverse: 5′-TGGAGATGTTGGTCAGGGAAT-3′; IL-33, forward: 5′- TCCAACTCCAAGATTTCCCCG-3′; IL-33, reverse: 5′-CATGCAGTAGACATGGCAGAA-3′; IL-5, forward: 5′-AGGAGCTTATTGAGGAGCTGA-3′; IL-5, reverse: 5′-TGGAGATGTTGGTCAGGG AAT-3′; Mcpt1, forward: 5′-TCCTGATGGCACTTCTCTTGC-3′; Mcpt1, reverse: 5′-TCCACTACAGTGTGCAGCAGT-3′; Mcpt6, forward: 5′-AGAACCAGGGCTGTGCTGTCT-3′; Mcpt6, reverse: 5′-AGAGGGAGCCACACAATGCAA-3′; GAPDH, forward: 5′-TCAACAGCAACTCCCACTCTT-3′; GAPDH, reverse: 5′-ACCCTGTTGCTGTAGCCGTAT-3′.

### Statistical Analyses

Data are presented as mean values ± SEM, as analyzed using GraphPad Prism 9.0. Two-way ANOVA statistical analysis was conducted to compare the *in vivo* lung function measurements between the experimental groups in response to the methacholine dose–response curve using the Bonferroni post-hoc analysis. For the rest of the experimental endpoints, one-way ANOVA was performed for statistical comparison between the treatment groups using Tukey post-hoc multi-comparison analysis. Differences between the experimental groups were regarded statistically significant when p-values reached ≤ 0.05.

## Results

### Intraperitoneally Administered Monensin has Dampening Effects on HDM-Induced Allergic Airway Inflammation

**Fig. 1:**
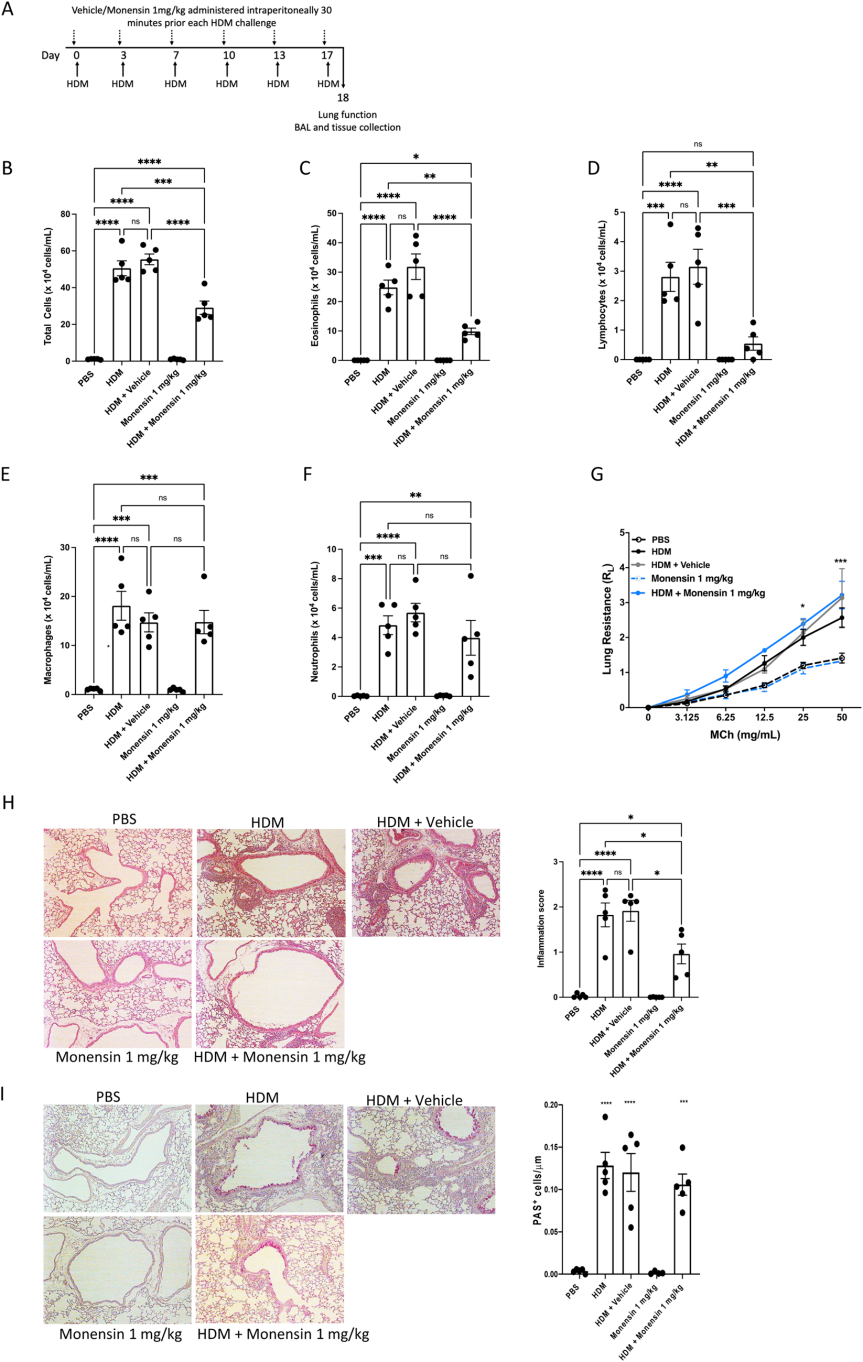
Monensin inhibits airway inflammation but not AHR in HDM-induced experimental asthma when administered intraperitoneally. **A** Mice received either PBS or HDM extract twice a week for 6 weeks. Mice were treated with vehicle or with monensin (1 mg/kg) intraperitoneally 30 min prior to each HDM instillation. Control mice were treated with PBS only. The number of total cells **B**, eosinophils **C**, lymphocytes **D**, macrophages **E** and neutrophils **F** in the bronchoalveolar lavage fluid were quantified. **G** Lung resistance (R_L_) was measured using a Buxco FinePointe series instrument. **H** Tissue inflammation was assessed after staining of lung sections with hematoxylin and eosin (H&E); quantification of tissue inflammation by scoring is shown to the right. **I** Goblet hyperplasia was quantified by PAS staining of lung sections; quantification of PAS^+^ cells is shown to the right. Data represent mean values ± SEM. *P ≤ 0.05, **P ≤ 0.01, ***P ≤ 0.001 and ****P ≤ 0.0001 vs. comparative control groups. N = 5 mice per group. HDM = house dust mite, ns = non significant

To assess the ability of monensin to dampen allergic airway responses, we first tested its effects following administration by the intraperitoneal (i.p.) route (protocol depicted in Fig. 1A). These analyses revealed that monensin caused a profound reduction in the total infiltration of cells into the airway lumen of HDM-sensitized mice (Fig. 1B). The reduction in total cell infiltration was mostly attributed to a substantial reduction in the numbers of eosinophils and lymphocytes, whereas the macrophage- and neutrophil populations were not significantly affected (Fig. 1C-F). Monensin was also shown to dampen the tissue inflammation, as manifested by a significant reduction in the inflammatory score (Fig. 1H). As seen in Fig. 1I, the HDM sensitization caused a profound goblet cell hyperplasia, as assessed by periodic acid-Schiff (PAS) staining. However, i.p.-administered monensin did not cause any significant effect on the goblet cell populations (Fig. 1I).

We also assessed whether i.p.-administered monensin could affect the airway hyperreactivity (AHR). AHR was assessed by measuring lung resistance (R_L_) in response to nebulized metacholine at increasing doses. As expected, the HDM sensitization produced a significant increase in AHR in response to methacholine. However, the AHR amplitude was not statistically different in vehicle- vs. monensin-treated mice that had been sensitized with HDM antigen, indicating that monensin has a limited capacity to block AHR when administered i.p. (Fig. 1G). Control experiments revealed that i.p.-administered monensin alone (in the absence of HDM sensitization) had no significant effect on airway inflammation and did not affect basal airway responses to methacholine (Fig. 1B-G).

### Effect of Intranasally Administered Monensin on AHR in HDM-Sensitized mice

**Fig. 2:**
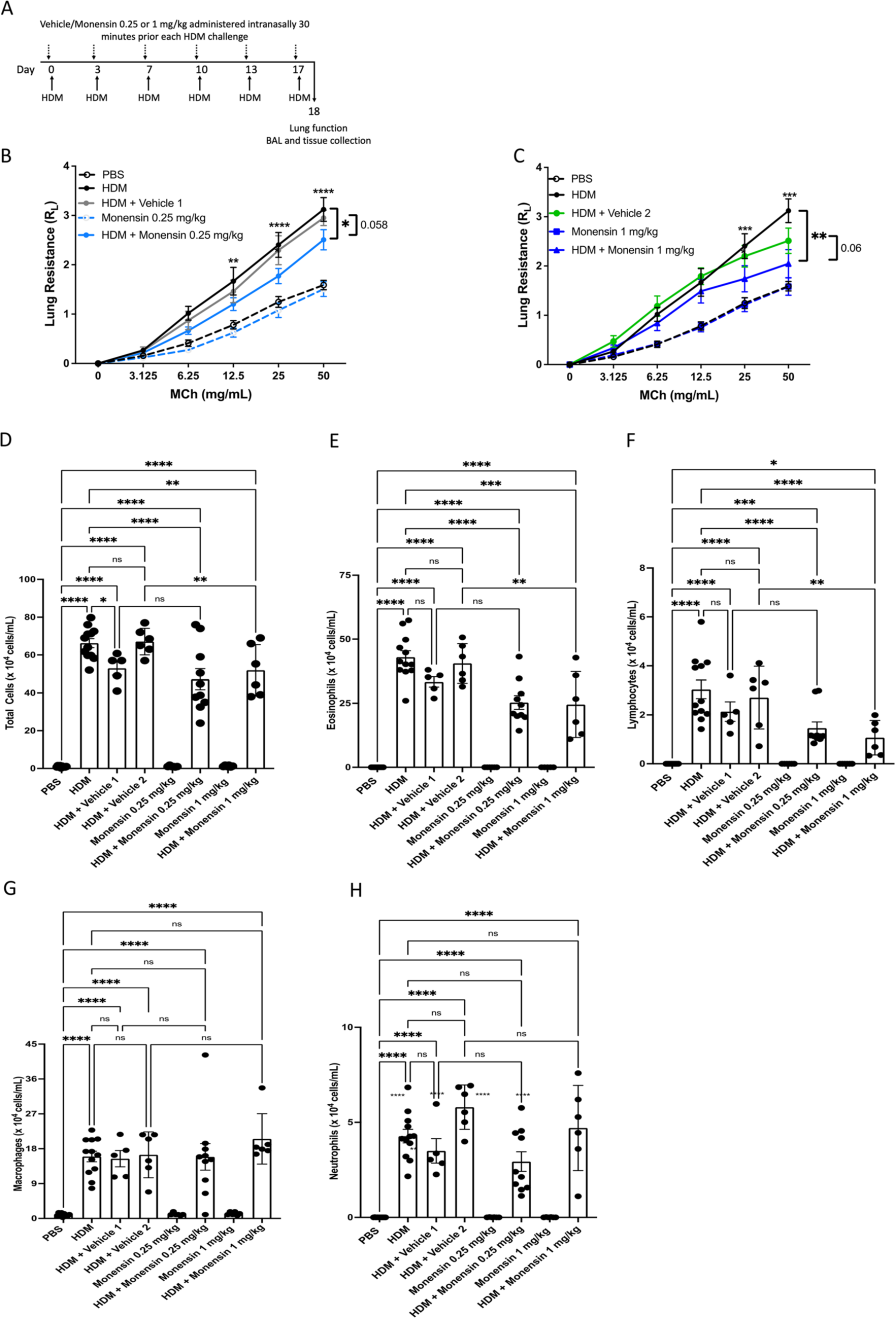
Monensin diminishes airway inflammation and AHR in HDM-induced experimental asthma when administered intranasally. **A** Mice received either PBS or HDM extract twice a week for 6 weeks. Mice were treated with vehicle (Vehicle 1 (4% ethanol in PBS) or Vehicle 2 (16% ethanol in PBS)) or with Monensin (0.25 or 1 mg/kg) 30 min intranasally prior to each HDM instillation. **B**-**C** Lung resistance (R_L_) was measured using a Buxco FinePointe series instrument. The number of total cells **D**, eosinophils **E**, lymphocytes **F**, macrophages **G**, and neutrophils **H** in the bronchoalveolar lavage fluid were quantified. Data represent mean values ± SEM. *P ≤ 0.05, **P ≤ 0.01, ***P ≤ 0.001 and ****P ≤ 0.0001 vs. comparative control groups. N = 6 –10 mice per group. HDM = house dust mite, ns = non significant

Since the data above indicate that systemic (i.p.) administration of monensin has a limited capacity to prevent allergen-induced AHR, we next assessed whether locally administered monensin could be more effective for this purpose. To this end, we administered monensin intranasally, at either a low dose (0.25 mg/kg) or high dose (1 mg/kg), followed by measurements of AHR (protocol shown in Fig. 2A).

As seen in Fig. 2B-C, the AHR was not affected by the vehicle used for monensin administration. Further, the administration of low-dose monensin caused a trend of suppressed AHR (HDM/vehicle vs. HDM/vehicle + monensin 0.25 mg/kg), although this did not reach statistical significance. A trend of suppressed AHR was also seen after administration of high-dose monensin (HDM/vehicle vs. HDM/vehicle + monensin 1 mg/kg)(Fig. 2C). Notably though, when comparing the HDM group with the HDM/vehicle + monensin groups (0.25 or 1 mg/kg/day), significantly suppressed AHR was seen (Fig. 2B-C).

### Intranasally Administered Monensin has Anti-Inflammatory Effects in HDM-Sensitized Mice

The effect of intranasally administered monensin on inflammatory parameters was also addressed. These assessments revealed that monensin administered both at the low and high dose caused a significant reduction of total cellular infiltration into the airway lumen in response to HDM sensitization (Fig. 2D). As for the i.p. route, this was mostly attributed to a profound reduction in the infiltration of eosinophils and lymphocytes, whereas the macrophage- and neutrophil populations were not significantly affected (Fig. 2E-H). The vehicles used for administration of monensin, either at the low or high doses, did not have any significant effects on the infiltration of any of the assessed cell populations, although a significant reduction in total number of cells was seen in response to vehicle 1 (Fig. 2D-H).

**Fig. 3:**
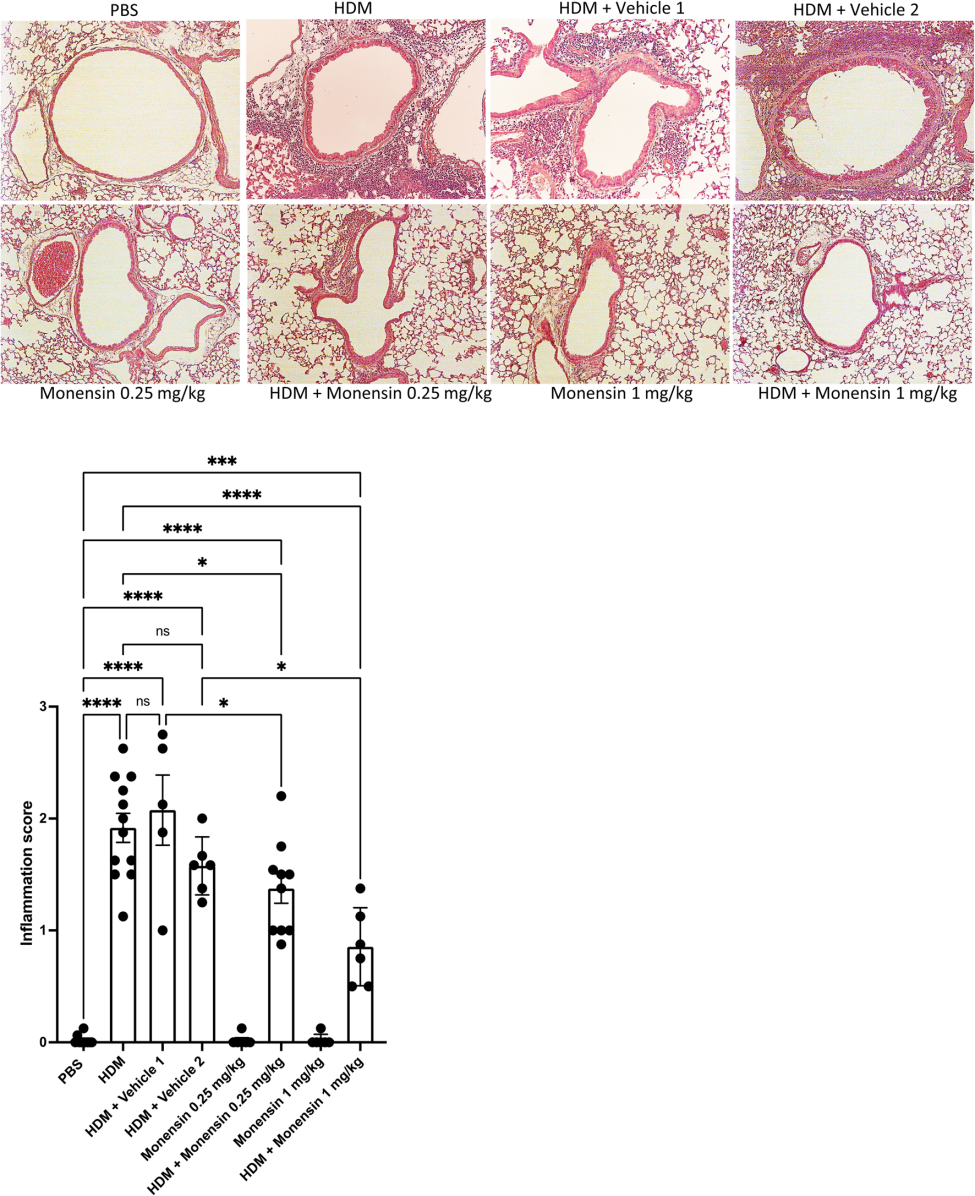
Monensin attenuates tissue inflammation in HDM-induced experimental asthma when administered intranasally. Mice received either PBS or HDM twice a week for 6 weeks. Mice were treated with vehicle (Vehicle 1 (4% ethanol in PBS) or Vehicle 2 (16% ethanol in PBS)) or with monensin (0.25 or 1 mg/kg) 30 min intranasally prior to each HDM instillation. Peribronchial inflammation around the airways was assessed by hematoxylin and eosin (H&E) staining of lung sections. Representative images for H&E (× 20 original magnification) are shown; quantification of tissue inflammation by scoring is depicted. Data represent mean values ± SEM. *P ≤ 0.05, ***P ≤ 0.001 and ****P ≤ 0.0001 vs. comparative control groups. N = 6 –10 mice per group. HDM = house dust mite, ns = non significant

The effect of intranasally administered monensin on lung tissue inflammation was also assessed. Firstly, these assessments confirmed that the HDM sensitization caused a profound inflammation of the lung tissue (Fig. 3). Further, these analyses showed that monensin at the low dose (0.25 mg/kg) caused a significant reduction in tissue inflammation, whereas the vehicle used for low dose monensin administration had no significant effect. Significant dampening of tissue inflammation was also seen in response to monensin administered at the high dose (1 mg/kg). Notably, a trend of reduced inflammation was also seen in response to the vehicle used for the high dose monensin administration. However, this did not reach statistical significance.

### Intranasally Administered Monensin Suppresses the Goblet Cell Population in HDM-Sensitized Mice

**Fig. 4:**
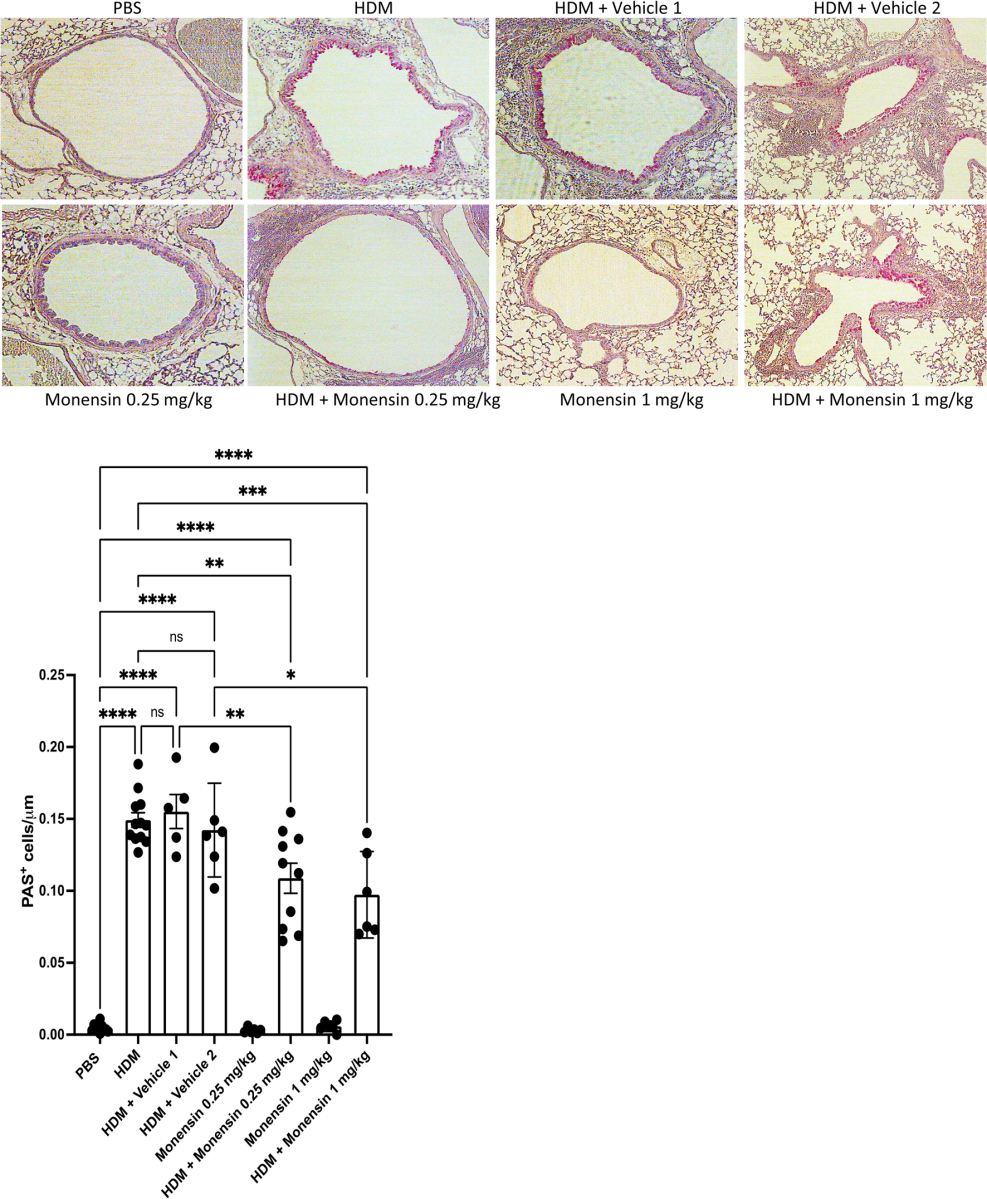
Monensin attenuates goblet hyperplasia in HDM-induced experimental asthma when administered intranasally. Mice received either PBS or HDM twice a week for 6 weeks. Mice were treated with vehicle (Vehicle 1 (4% ethanol in PBS) and Vehicle 2 (16% ethanol in PBS)) or with monensin (0.25 or 1 mg/kg) 30 min intranasally prior to each HDM instillation. Goblet hyperplasia around the airways in the lung sections was assessed by PAS staining. Representative images for PAS (× 40 original magnification) are shown; quantification of PAS^+^ cells is depicted. Data represent mean values ± SEM. *P ≤ 0.05, ***P ≤ 0.001 and ****P ≤ 0.0001 vs. comparative control groups. N = 6 –10 mice per group. HDM = house dust mite, ns = non significant

To assess whether intranasal administration of monensin affects the airway goblet cell populations, PAS staining of lung sections was performed. These analyses revealed that the HDM sensitization caused a marked increase in the PAS^+^ cell population, in agreement with an expansion of the goblet cell population (Fig. 4). Further, it was seen that that monensin, both at the low and high dose, caused a significant reduction in the numbers of PAS^+^ cells (Fig. 4). Hence, these data indicate that intranasally administered monensin has the capacity to suppress the expansion of the goblet cell populations that occur in response to allergen sensitization.

### Effect of Intranasally Administered Monensin on the Thickening of the Airway Smooth Muscle Layer in HDM-Sensitized Mice

**Fig. 5:**
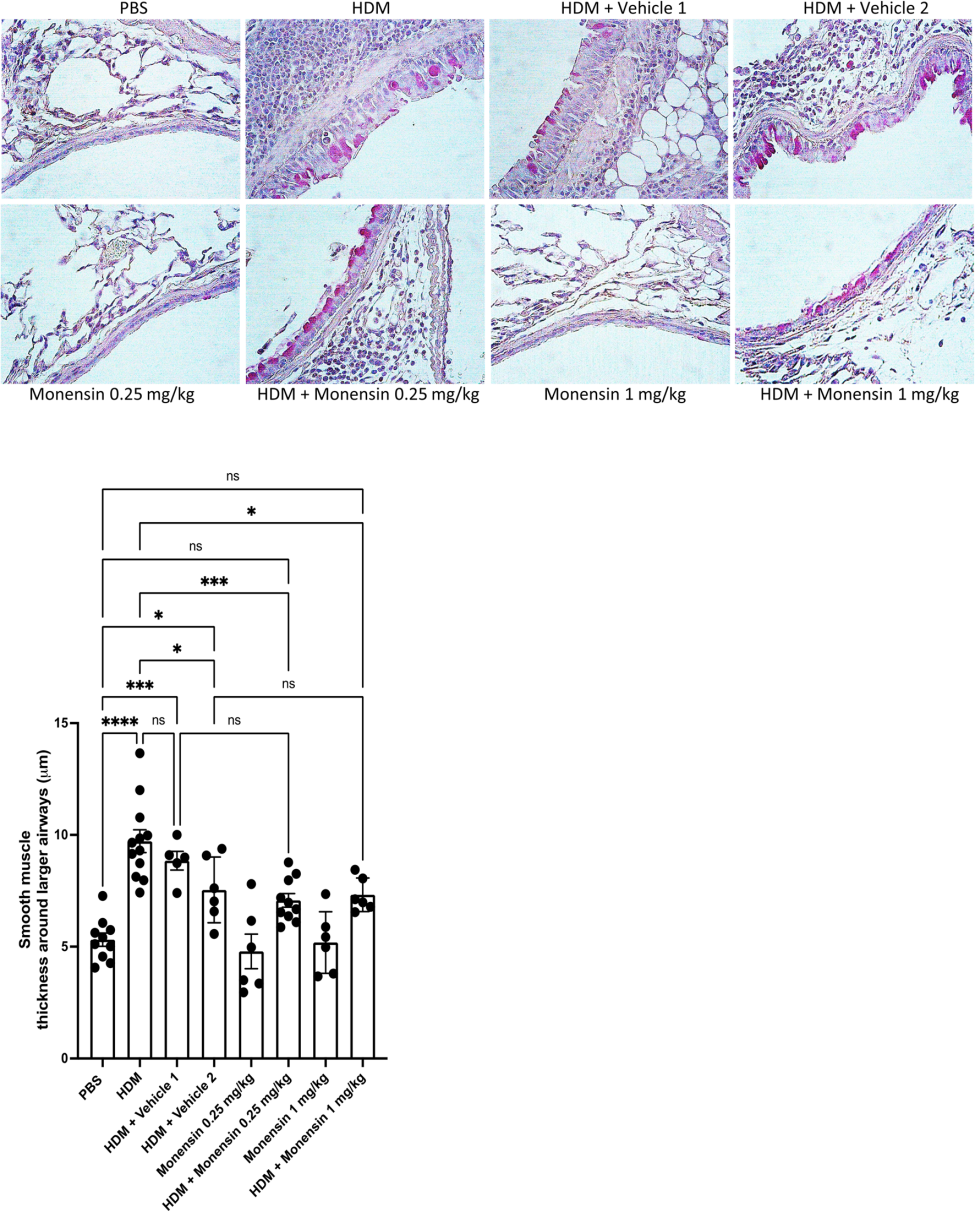
Monensin reduces airway smooth muscle thickness in HDM-induced experimental asthma when administered intranasally. Mice received either PBS or HDM extract twice a week for 6 weeks. Mice were treated with vehicle [Vehicle 1 (4% ethanol in PBS) or Vehicle 2 (16% ethanol in PBS)] or with monensin (0.25 or 1 mg/kg) 30 min intranasally prior to each HDM instillation. Smooth muscle thickness around the airways in the lung sections was assessed by PAS staining. Representative images for PAS staining (× 40 original magnification) are shown; quantification of smooth muscle layer thickness is depicted. Data represent mean values ± SEM. *P ≤ 0.05, ***P ≤ 0.001 and ****P ≤ 0.0001 vs. comparative control groups. N = 6 –10 mice per group. HDM = house dust mite, ns = non significant

Thickening of the airway smooth muscle layer is a hallmark event occurring in allergic asthma, and in the next set of experiments we assessed whether monensin can affect this process. As seen in Fig. 5, the HDM sensitization procedure, as expected, led to a significant thickening of the airway smooth muscle cell layer. This was seen when comparing the PBS group with both the HDM- and HDM/vehicle groups. In contrast, no significant increase in smooth muscle layer thickening was seen when comparing the PBS group with the HDM/monensin groups (high or low dose), in agreement with a dampening effect of monensin on HDM-induced airway smooth muscle cell layer thickening.

### Intranasally Administered Monensin Suppresses MC Degranulation in HDM-Sensitized Mice

**Fig. 6:**
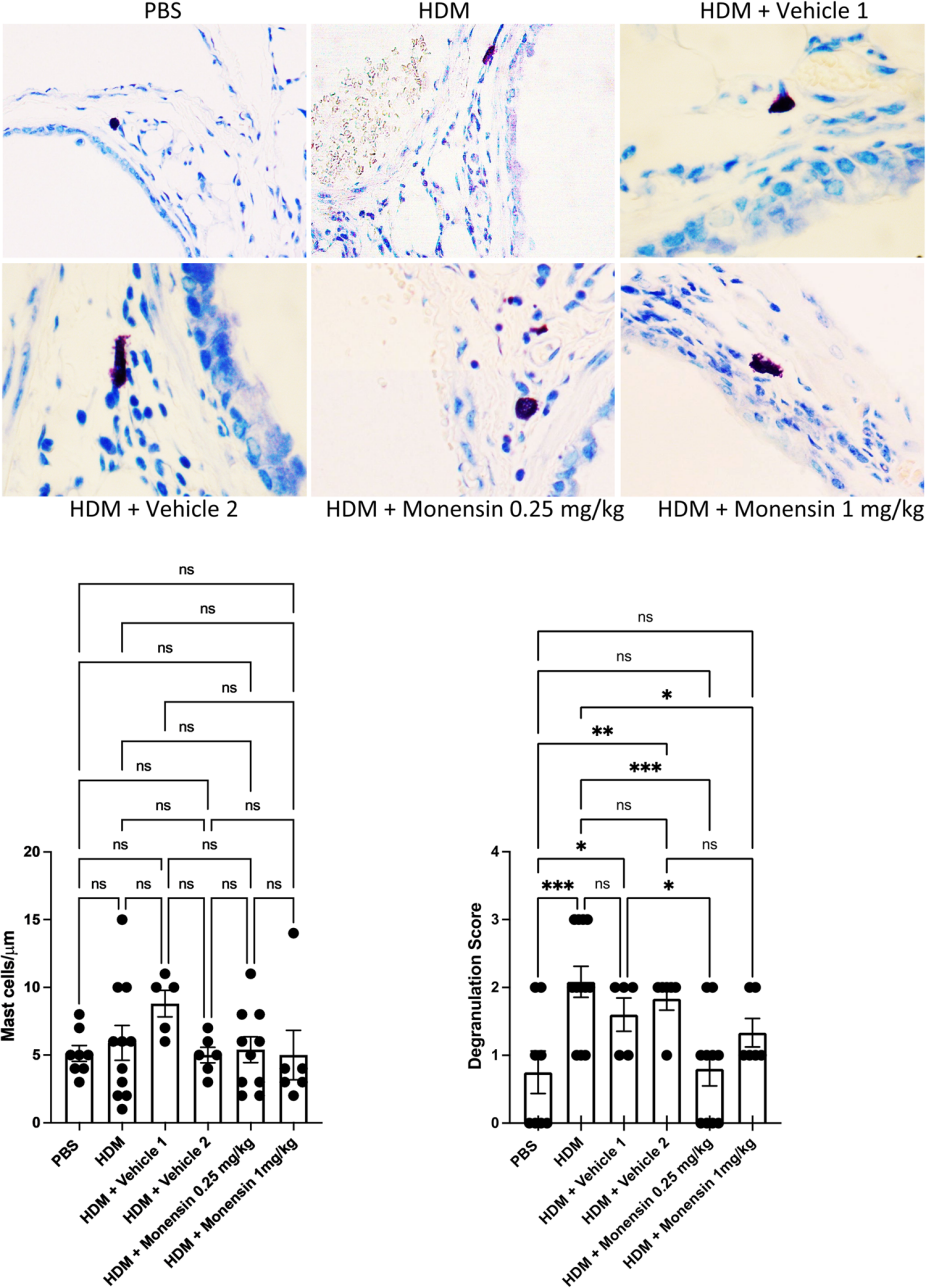
Monensin inhibits MC degranulation without affecting MC numbers in HDM-induced experimental asthma. Mice received either PBS or HDM extract twice a week for 6 weeks. Mice were treated with monensin (0.25 or 1 mg/kg) 30 min intranasally prior to each HDM instillation. MC numbers and MC degranulation around the larger airways was assessed by toluidine blue staining of lung sections. Representative images for toluidine blue staining (× 40 original magnification) are shown; quantification of MC numbers and extent of MC degranulation is depicted. Data represent mean values ± SEM. *P ≤ 0.05, **P ≤ 0.01, and ***P ≤ 0.001 vs. comparative control groups. N = 6 –10 mice per group. HDM = house dust mite, ns = non significant

Since monensin has previously been shown to have potent anti-MC activities [21], we next asked whether monensin exerts such effects on MC populations in the context of HDM-induced allergic airway inflammation. To this end, tissue sections from lungs of HDM-sensitized mice were stained with toluidine blue, followed by the quantification of MC numbers and extent of MC degranulation. As seen in Fig. 6, MCs were predominantly seen around the larger airways. No effect of monensin was seen on the total numbers of lung MCs. However, the low dose of monensin caused a significant reduction in the number of degranulated (activated) MCs (Fig. 6).

### Intranasally Administered Monensin Modifies the Expression of CCL11, CCL9 and Clec7a in Lungs of HDM-Sensitized Mice

**Fig. 7:**
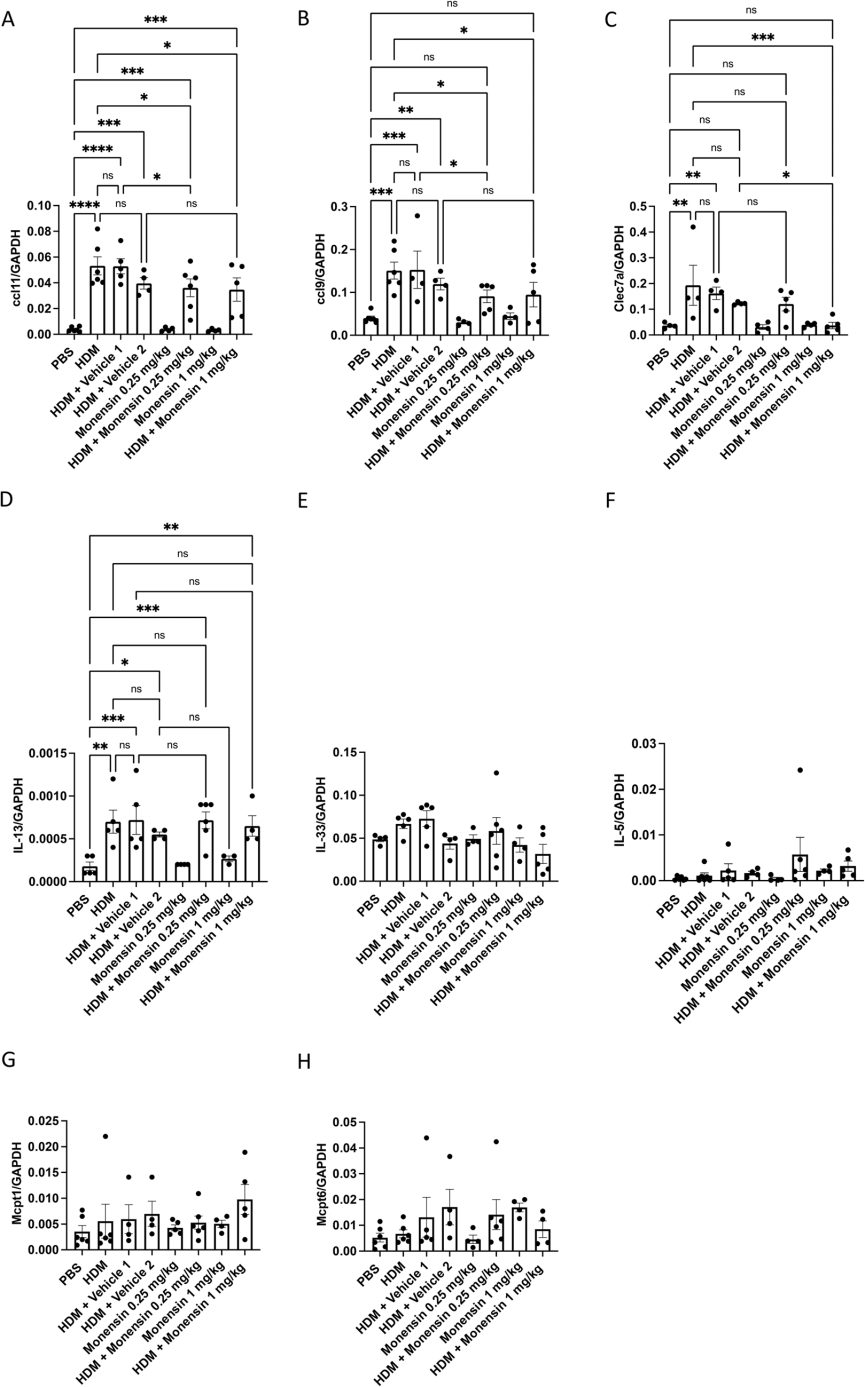
Monensin reduces the expression of CCL11 and CLL9 and Clec7a/Dectin1 in HDM-induced experimental asthma. Mice received either PBS or HDM extract twice a week for 6 weeks. Mice were treated with monensin (0.25 or 1 mg/kg) 30 min intranasally prior to each HDM instillation. Total RNA was recovered from the lungs and was assessed by qPCR analysis for levels of mRNA coding for: CCL11(**A**), CCL9 (**B**), Clec7a/Dectin1(**C**), IL-13 (**D**), IL-33(**E**), IL-5 (**F**), Mctp1 (**G**) and Mctp6 (**H**). Data was normalized to GAPDH expression. Data represent mean values ± SEM. Data represent mean values ± SEM. *P ≤ 0.05, **P ≤ 0.01, ***P ≤ 0.001 and ****P ≤ 0.0001 vs. comparative control groups. N = 4 – 6 mice per group. HDM = house dust mite, ns = non significant

To provide further insight into the mechanism by which monensin ameliorates features of allergic airway inflammation in response to HDM sensitization, we assessed whether monensin affects the expression of genes coding for selected inflammatory markers: CCL11, CCL9, Clec7a/Dectin-1, IL-13, IL-33 and IL-5. As depicted in Fig. 7A-7D, HDM sensitization caused a profound increase in the expression of the CCL11, CCL9, Clec7a/Dectin-1 and IL-13 genes, the latter in clear agreement with HDM sensitization causing a Th2 response. In contrast, no significant upregulation of either IL-33 or IL-5 gene expression was seen (Fig. 7E-F). Further, it was noticed that intranasally administered monensin caused a significant reduction in the expression of the CCL11, CCL9 and Clec7a genes (Fig. 7A-C).

Since data presented here and elsewhere [21] have revealed that MCs are affected by monensin, we also assessed for effects of monensin on the expression of MC markers. To this end, we measured the expression of the mucosal-type MC marker Mcpt1, and the expression Mcpt6, Mcpt6 being known to be expressed both by classical connective tissue-type MCs as well as by mucosal MC populations of the lung [28]. These analyses showed that both Mcpt1 and Mcpt6 were expressed in the airways (Fig. 7G-H). However, the HDM sensitization did not affect the expression of these genes, and the levels of Mcpt1 and Mcpt6 expression were not significantly influenced by monensin (Fig. 7G-H).

## Discussion

Previous research has established MCs as emerging targets for treatment in various inflammatory settings, in particular of allergic type, and various strategies for this purpose are currently available [17–19]. A major challenge for such initiatives is to identify regimes that show selectivity for MCs. To achieve this, it is necessary to focus on unique features of MCs, and in our previous efforts we have reasoned that MCs may be selectively sensitive to strategies that target their secretory granules, based on the notion that MCs have a uniquely high content of secretory granules filled with potentially toxic compounds [15]. Indeed, we have previously shown that MCs are highly sensitive to a variety of compounds having in common that they can permeabilize the MC secretory granules, leading to efflux of granule content into the cytosol [17, 29–34]. One of these compounds is monensin, which was identified as a potent anti-MC compound through a drug repurposing approach [21]. In agreement with its anti-MC effects, it has recently been demonstrated that monensin has the capacity to dampen AHR in a guinea pig model of asthma, and also that monensin can block airway constriction *ex vivo* in human lung specimens [23]. Further, it was recently demonstrated that monensin, in addition to its anti-MC effects, also can target eosinophils [22]. Notably, eosinophils have several features in common with MCs, e.g., a high content of acidic secretory granules, and our findings revealed that monensin targets eosinophils through similar mechanisms as those operative in its effects on MCs [22].

Here we evaluated the effect of monensin in a physiologically relevant mouse model for allergic airway inflammation, based on sensitization with a major allergen (HDM antigen). Our findings reveal that monensin has the capacity to dampen multiple pathological features in this model. Importantly, no overall signs of toxicity in response to monensin were observed. Hence, the current study provides strong support for the notion that monensin may be a promising candidate for usage in anti-asthma medication.

Our findings reveal that monensin had profound anti-inflammatory effects both after systemic (i.p.) administration and after local installation (intranasally). The exact mechanism behind the impact of monensin on features of allergic airway inflammation is intriguing. Since our previous findings have shown that monensin has potent anti-MC properties, a likely scenario is that the effects of monensin in the HDM model of asthma is, at least partly, due to its effects on MC populations. In line with such a scenario, MCs have been shown to be important for the development of allergic airway inflammation and AHR in response to HDM sensitization in mice [35].

In agreement with an effect of monensin on the lung MC population, it was noted that monensin caused a profound decrease in the extent of MC degranulation seen in response to HDM provocation. Hence, a likely scenario is that the dampening of airway responses by monensin is linked to a reduced release of pro-inflammatory mediators from MCs. However, considering that monensin previously has been shown to induce MC apoptosis [21], we would have expected to see a reduction in MC numbers in response to monensin administration. Possibly, these finding might thus suggest that monensin, in addition to its pro-apoptotic impact on MCs, may have the capacity to interfere with MC degranulation under conditions prevailing in the lungs of HDM-sensitized mice.

A major finding in this study was that eosinophil numbers were profoundly decreased after monensin administration. Potentially, this could be due to direct pro-apoptotic effects of monensin on eosinophil populations, which would be in agreement with previous findings [22]. However, we cannot exclude that the observed effects on eosinophil infiltration could be due to indirect effects. Notably, monensin was shown to cause a downregulation of CCL11 (eotaxin-1), a key factor in the recruitment of eosinophils [36]. It is thus possible that the suppression of eosinophil infiltration in response to monensin might be linked, a least partly, to downregulated expression of this chemokine.

Another intriguing finding in this study was that monensin had a major impact on the infiltration of lymphocytes into the airway lumen of HDM-sensitized animals. In our previous studies, we did not note any direct cytotoxic impact of monensin on lymphocyte populations [21], suggesting that the reduction of lymphocytes seen in this study may be explained by indirect effects of monensin.

When assessing for effects on the expression of inflammatory markers, we noticed that monensin suppressed the expression of the gene coding for Clec7a/Dectin-1. Interestingly, Clec7a/Dectin-1 has previously been shown to be induced under allergic airway inflammatory conditions [24, 37] and, intriguingly, it has been shown that Clec7a/Dectin-1 expression is linked to the expansion of Cd11b^+^ dendritic cells [37]. Hence, the suppression of Clec7a expression by monensin could potentially lead to a reduction in the population of Cd11b^+^ dendritic cells, which, in turn, could lead to an impaired adaptive immune response. In line with this, Cd11b^+^ dendritic cells have been shown to be crucial for the immune responses after sensitization with HDM antigen [38]. We could thus speculate that the reduction in lymphocyte numbers following monensin instillation could, at least partly, be due to effects on adaptive immune mechanisms. However, extended studies are warranted to define the exact molecular mechanism underlying these findings.

## Data Availability

Data will be made available on request.

## Abbreviations

HDM: House dust mite
AHR: Airway hyperreactivity
MC: Mast cell
BAL: Bronchoalveolar lavage
PAS: Periodic acid-Schiff
